# A mouse anti-myostatin antibody increases muscle mass and improves muscle strength and contractility in the mdx mouse model of Duchenne muscular dystrophy and its humanized equivalent, domagrozumab (PF-06252616), increases muscle volume in cynomolgus monkeys

**DOI:** 10.1186/s13395-017-0141-y

**Published:** 2017-11-09

**Authors:** Michael St. Andre, Mark Johnson, Prashant N. Bansal, Jeremy Wellen, Andrew Robertson, Alan Opsahl, Peter M. Burch, Peter Bialek, Carl Morris, Jane Owens

**Affiliations:** 1 Rare Disease Research Unit, Pfizer Inc., 610 Main Street, Cambridge, MA 02139 USA; 2 NIGMS Training Program in Biomolecular Pharmacology, Department of Pharmacology & Experimental Therapeutics, Boston University School of Medicine, Boston, MA USA; 3 Early Clinical Development, Pfizer Inc., Cambridge, MA USA; 4 Investigative Pathology, Pfizer Inc., Groton, CT USA; 5 Research and Development Drug Safety, Pfizer Inc., Groton, CT USA; 6 Present Address: PAREXEL Informatics, Billerica, MA USA; 7 Present Address: Summit Therapeutics, Cambridge, MA USA; 8 Present Address: Solid Biosciences, Cambridge, MA USA; 9 Present Address: Proteostasis Therapeutics, Cambridge, MA USA

**Keywords:** Myostatin, Hypertrophy, Skeletal muscle, Duchenne muscular dystrophy, Monoclonal antibody, mdx

## Abstract

**Background:**

The treatments currently approved for Duchenne muscular dystrophy (DMD), a progressive skeletal muscle wasting disease, address the needs of only a small proportion of patients resulting in an urgent need for therapies that benefit all patients regardless of the underlying mutation. Myostatin is a member of the transforming growth factor-β (TGF-β) family of ligands and is a negative regulator of skeletal muscle mass. Loss of myostatin has been shown to increase muscle mass and improve muscle function in both normal and dystrophic mice. Therefore, myostatin blockade via a specific antibody could ameliorate the muscle weakness in DMD patients by increasing skeletal muscle mass and function, thereby reducing patients’ functional decline.

**Methods:**

A murine anti-myostatin antibody, mRK35, and its humanized analog, domagrozumab, were developed and their ability to inhibit several TGB-β ligands was measured using a cell-based Smad-activity reporter system. Normal and *mdx* mice were treated with mRK35 to examine the antibody’s effect on body weight, lean mass, muscle weights, grip strength, ex vivo force production, and fiber size. The humanized analog (domagrozumab) was tested in non-human primates (NHPs) for changes in skeletal muscle mass and volume as well as target engagement via modulation of circulating myostatin.

**Results:**

Both the murine and human antibodies are specific and potent inhibitors of myostatin and GDF11. mRK35 is able to increase body weight, lean mass, and muscle weights in normal mice. In *mdx* mice, mRK35 significantly increased body weight, muscle weights, grip strength, and ex vivo force production in the extensor digitorum longus (EDL) muscle. Further, tibialis anterior (TA) fiber size was significantly increased. NHPs treated with domagrozumab demonstrated a dose-dependent increase in lean mass and muscle volume and exhibited increased circulating levels of myostatin demonstrating target engagement.

**Conclusions:**

We demonstrated that the potent anti-myostatin antibody mRK35 and its clinical analog, domagrozumab, were able to induce muscle anabolic activity in both rodents, including the mdx mouse model of DMD, and non-human primates. A Phase 2, potentially registrational, clinical study with domagrozumab in DMD patients is currently underway.

## Background

Duchenne muscular dystrophy (DMD) is characterized by progressive skeletal muscle wasting and weakness leading to loss of ambulation and premature death from respiratory and cardiac failure [1]. There are only two approved therapies for DMD patients, Translarna (ataluren, PTC Therapeutics) and Exondys 51 (eteplirsen, Sarepta), and both received conditional approvals in the European Union (EU) and United States of America (USA), respectively, which means additional clinical trials are required to verify their predicted clinical benefit [2–6]. Furthermore, only a limited number of DMD patients may benefit from these mutation-specific therapies. Translarna and Exondys 51 treatments are designed to address the needs of only 13 and 14% of the DMD patient population, respectively, leaving a large number of patients without treatment [7, 8]. Therefore, an unmet need remains for patients affected by this debilitating disease. Our focus has been to target ameliorating muscle weakness in DMD patients by increasing skeletal muscle mass and strength, thereby reducing their functional decline. This treatment could benefit any patient with DMD, regardless of the underlying mutation. We aim to do this by inhibiting myostatin activity in skeletal muscle.

Myostatin, originally termed growth differentiation factor-8 (GDF-8), is a member of the transforming growth factor-β (TGF-β) superfamily and a negative regulator of skeletal muscle mass [9]. Naturally occurring null mutations in the myostatin gene of cows, sheep, dogs, and pigs result in hyper-muscular phenotypes, and genetic ablation of myostatin in mice has resulted in a similar muscle phenotype [10, 11]. Importantly, racing whippet dogs carrying at least one mutant myostatin allele displayed enhanced racing performance compared with normal whippets [12], providing evidence that disruption in the expression and function of myostatin resulted in a functional benefit in these dogs.

Like all members of the TGF-β superfamily, myostatin signals through a heteromeric complex of serine/threonine kinase receptors, which transduces signals to the nucleus through intracellular proteins called Smads. Myostatin first binds to its high-affinity receptor, activin receptor type IIB (ActRIIB), which then recruits and phosphorylates the low-affinity receptor, activin receptor like kinase 4 or 5 (Alk4 or Alk5). The low-affinity receptor signals by phosphorylating Smad2 or Smad3, which forms a complex with Smad4 that enters the nucleus to induce transcriptional activities [13]. The end result of this signaling pathway is inhibition of myogenic processes in muscle precursor (satellite) cells and in mature, differentiated muscle fibers.

Given its negative effect on skeletal muscle mass, myostatin represents an attractive target for the treatment of diseases associated with muscle loss, including DMD. In addition to the naturally occurring myostatin mutations and genetic myostatin ablation models, there is evidence for a beneficial effect in skeletal muscle in response to pharmacological inhibition of myostatin in both wild-type and mdx mice, a mouse model of DMD [14]. Myostatin inhibition has been demonstrated with several biotherapeutic modalities including anti-myostatin antibodies, a myostatin propeptide, a soluble ActRIIB-Fc, and antisense oligonucleotides that block signaling activity [15–20]. Studies with each of these targeting strategies have shown increased skeletal muscle mass and improved muscle function in both normal and dystrophic animals. Our construct mRK35 is a novel mouse monoclonal antibody that inhibits myostatin signaling by binding to the mature myostatin dimer to block its interaction with ActRIIB, thereby inhibiting the initiation of the Smad2/3 signaling pathway [21]. A humanization campaign to generate an equivalent human anti-myostatin antibody has resulted in the generation of the humanized IgG1 monoclonal antibody domagrozumab, PF-06252616.

Previous evaluation of mRK35 in rodent models of ALS demonstrated its capacity to increase skeletal muscle mass and strength [22]. Here, we present studies with mRK35 that demonstrate its efficacy in wild-type mice and in the mdx mouse model of DMD at increasing skeletal muscle mass and function. Additionally, we report studies with the humanized anti-myostatin antibody domagrozumab in non-human primates that demonstrate increased skeletal muscle mass and volume following 8 weeks of weekly dosing, thereby providing further justification for progression of this molecule to drug safety studies and clinical testing.

## Methods

### Reagents and antibodies

Myostatin was expressed from a Chinese hamster ovary (CHO) cell line and purified using methods similar to those described in Thies et al. (2001) [23]. The construction, expression, and purification of ActRIIB-FC were previously described in Lee et al. [24]. Recombinant human GDF-11, Activin A, Activin B, Activin AB, and BMP-9 (bone morphogenetic protein-9) were purchased from R&D Systems (Minneapolis, MN, USA). Murine RK35 (mRK35) is a mouse monoclonal antibody against mature myostatin. It was generated from myostatin knockout mice immunized with human myostatin. This antibody was selected for humanization as described in Apgar et al. [21], resulting in the generation of domagrozumab.

### Cell-based activity assays

Myostatin activity was measured using A204 cells transfected with the pGL3-(CAGA)_12_-luciferase reporter as described previously [23]. A204-CAGA_12_-luciferase cells (30,000/well) were cultured in 96 well plates for 16 h at 37 °C in McCoy’s 5A media supplemented with 1 mM glutamine, 100 U/mL streptomycin, 100 μg/mL penicillin, and 10% fetal bovine serum. Activation of signaling was induced with 1 nM myostatin (pre-determined EC_50_ concentration) incubated with the cells for 6 h at 37 °C. To examine the inhibitory activity of test compounds, 1 nM of myostatin was pre-incubated with a doubling dilution series (starting at ~ 6 nM) of the test compounds for 30 min at room temperature in McCoy’s 5A medium (as described above), except serum was replaced with 1 mg/mL BSA before adding the mixture to the cell plates and incubating at 37 °C for a further 6 h. Luciferase activity (luminescence) was measured using BriteLite Plus (Perkin Elmer, Waltham, MA, USA). This assay was also used to examine the selectivity of test compounds against other TGF-β family ligands including GDF-11, Activin A, Activin B, and Activin AB using their EC_50_ concentrations (0.3, 0.4, 0.2, and 1 nM, respectively) to activate signaling.

The selectivity of test compounds was further assessed in a BMP-induced reporter gene assay (RGA). For this assay, BMP-9 activity was measured using C2C12 mouse myoblast cells transfected with a pTal-Luc reporter plasmid in which a synthetic BMP-response element (BRE) was inserted into the NheI site as previously described [25]. C2C12-BRE-Luc cells (10,000/well) were cultured in 96 well plates for 16 h at 37 °C in low bicarbonate Dulbecco’s Modified Eagle’s Medium (DMEM) supplemented with 4 mM L-glutamine, 4.5 g/L glucose, 100 U/mL streptomycin, 100 μg/mL penicillin, and 10% fetal bovine serum. Activation of signaling was induced with 0.03 nM BMP-9 (pre-determined EC_50_ concentration) incubated with the cells for 16 h at 37 °C. To examine the inhibitory activity of test compounds, 0.03 nM of BMP-9 was pre-incubated with a doubling dilution series (starting at ~ 6 nM) of the test compounds for 30 min at room temperature in DMEM (as described above), except with 1% serum, before adding the mixture to the cell plates and incubating at 37 °C for a further 16 h. Luciferase activity (luminescence) was measured using BriteLite Plus (Perkin Elmer, Waltham, MA, USA).

### In vivo studies in mdx mice

Male C57Bl/10ScSn-Dmd<mdx>/J and C57Bl/10J mice were purchased from the Jackson Laboratory (Bar Harbor, Maine, USA) and C57Bl/6 mice were purchased from Charles River Laboratories (Wilmington, MA, USA). All mice were housed in a facility with 12-h light-dark cycle and fed standard mouse diet (Lab Diet 5053 Lab Diet, St. Louis, MO, USA) and water ad libitum. All animal procedures were approved by the Institutional Animal Care and Use Committee (IACUC) and were carried out in an Association for Assessment and Accreditation of Laboratory Animal (AAALAC) accredited facility.

Starting at either 8 weeks of age or 1 year of age, mice were randomized by body weight and given weekly intraperitoneal (IP) injections of either 10 mg/kg mRK35 or vehicle (PBS). Body weights and body composition, as measured by a Bruker Optics MiniSpec TD-Nuclear Magnetic Resonance (NMR) (Billerica, MA, USA), were taken weekly, and forelimb grip strength was measured at the end of the study. After 8 weeks of treatment, mice were anesthetized with a ketamine/xylazine cocktail and the extensor digitorum longus (EDL) muscle was removed and placed in an oxygenated Ringer’s bath. Silk suture was tied to the proximal and distal ends of the EDL just beyond the myotendinous junction, and the muscle was suspended between the lever arm of a force transducer (Aurora Scientific, Aurora, ON, Canada) and an immobile post. The muscle was set to resting length (L_0_) by a series of twitches. An electrical pulse at 120 Hz for one-half second was applied to the muscle three times with a 2-min rest period between each pulse to elicit isometric tetanic contractions, and the maximum force was recorded. Cross-sectional area was calculated as the ratio of muscle mass to the product of mammalian muscle density (1.06 mg/mm^3^), the resting length L_0_, and 0.45 (a published muscle-to fiber-length constant [26]). Specific force was calculated as tetanic force normalized by cross-sectional area. The tibialis anterior (TA), gastrocnemius bundle, and quadriceps muscles were removed for wet weights.

### Muscle histology and morphometry

For fluorescent imaging, dissected TA muscles were positioned on glass coverslips and mounted on cork disks using optimal cutting temperature medium (Sakura Finetek, Torrance, CA USA) before snap freezing in liquid-nitrogen-cooled isopentane. The glass coverslip was removed and 10-μ transverse sections were cryosectioned, air dried, and fixed for 5 min with 4% paraformaldehyde in Dulbecco’s phosphate buffered saline (DPBS), pH 7.2 (DPBS). Sections were permeabilized with 0.1% Triton X-100 in DPBS for 5 min prior to staining for 4 h at room temperature with 5 μg/mL Alexa 488-labeled wheat-germ agglutinin (Thermo Fisher Inc. [Waltham, MA, USA]) diluted in DPBS. Sections were washed and mounted in Prolong Diamond with 4′,6-diamidino-2-phenylindole (DAPI) (Thermo Fisher Inc. [Waltham, MA, USA]). Representative images were captured with a Zeiss 710 laser scanning confocal microscope. For image analysis, slides were scanned using a Leica Ariol and automated myofiber cross-sectional area measurements of 1400–1800 myofibers per animal were conducted using Image-Pro Plus image analysis software (Media Cybernetics, Rockville, MD, USA).

### In vivo studies in non-human primates (NHPs)

Male cynomolgus monkeys were purchased from Covance Research (Princeton, NJ). All animals were maintained in a facility with a 12-h light-dark cycle and fed standard monkey chow (Purina LabDiet, PMI 5 K91 certified; PharmaServ, Framingham, MA, USA) and water ad libitum. All animal procedures were approved by the IACUC and were carried out in an AAALAC accredited facility.

Thirteen male NHPs (age, 3–5 years; weight, 3.3–5.4 kg) were randomized into three groups and administered weekly intravenous (IV) infusions for 8 weeks of either vehicle (*n* = 5), 10 mg/kg (low dose, *n* = 5) or 30 mg/kg (high dose, *n* = 3) of domagrozumab. Monkeys were chair-trained to allow for conscious dosing. Body weight was monitored weekly and doses were adjusted for changes in body weight. DXA measurements were recorded at baseline (week 0), week 4, 8, 12, 17, and 25 and X-ray CT imaging was conducted at weeks 0 and 8. In preparation for imaging sessions, fasted animals were pretreated with atropine and anesthetized with tiletamine-zolazepam. Anesthesia was maintained via delivery of isoflurane in 100% oxygen as the carrier gas. Serum was collected at baseline, during weeks 1 through 7 (prior to compound administration), and during the post-observation period at weeks 8, 10, 12, and 17.

### In vivo imaging

Whole-body dual energy X-ray absorptiometry (DXA) scans (Luna iDXA, GE Healthcare, Madison, WI, USA) were conducted by positioning anesthetized animals supine and centered on the scanner bed with the spine oriented along the long-axis centerline of the scanner table. Attention was paid to ensure consistent positioning of the arms and legs of each animal so as to maintain a constant spacing between torso and arms, and a triangular-shaped foam positioning guide was placed between the legs to keep the legs parallel to one another and flat on the scanner bed.

Following DXA scanning at weeks 0 and 8, animals were transferred to the computerized tomography (CT) scanning bed (CereTom, NeuroLogica, Danvers, MA, USA). As with the DXA scans, consistent animal body positioning was maintained through the use of positioning aids including foam blocks to achieve uniform spacing between the legs and between the torso and arms; additionally, elastic wrap was used around the legs and scanning bed at the level of the patella to keep the legs flat and parallel to one another. The CT acquisition protocol consisted of contiguous axial slices prescribed to extend from approximately the L2 vertebra to the distal-most end of the femur. All scans were acquired with a tube voltage of 120 kVp, tube current of 4 mA, 6-s integration time, 25 cm FOV, 0.625 mm slice thickness; standard image reconstruction was performed on the scanner using a soft-tissue kernel to yield images with approximately 0.5 mm × 0.5 mm in-plane resolution.

#### Image analysis

Analysis of DXA scans was conducted using standard output generated by analysis software provided with the scanner to derive values of muscle tissue mass for the lower limbs (i.e., combined muscle mass of both legs).

Analysis of CT scans was conducted off-line from the scanner using the commercial image processing software Analyze (AnalyzeDirect, Overland Park, KS, USA). The aim of the analysis was to quantify longitudinal change in muscle volume over the course of the study period. A brief summary of the processing steps followed in this analysis is provided here.

#### Appendicular muscle

Appendicular muscle volume of each leg was quantified at a mid-femur location that was consistently defined across time points. To accomplish this, sub-regions were extracted from the scan volume that encompasses each leg, and anatomical landmarks were identified that correspond with the most distal and most proximal ends of the femur’s intramedullary canal. These landmarks were used to spatially register each leg to its corresponding baseline dataset. A series of intensity thresholding and region-growing operations were then applied to segment the registered dataset by tissue type (i.e., intramedullary canal, bone, fat, and muscle). Finally, the volume of segmented leg muscle was calculated over an axial section of tissue that extended 2.5 cm distal to the mid-femur position.

#### Axial muscle

Axial muscle volume along the spinal column was quantified from a sub-region of the scan volume defined by the extent of axial slices that encompass the L3 vertebra. Similar to the appendicular muscle segmentation routine, a series of intensity thresholding and region-growing operations were applied to segment the sub-region by tissue type (i.e., bone, fat, and muscle) from which the volume of the resulting muscle segment was calculated.

### Bioanalytical method for the quantification of total myostatin in cynomolgus monkey serum

The concentrations of total myostatin in monkey serum were quantified using an enzyme-linked immunoassay (ELISA) developed by Pfizer. In the assay, myostatin was captured onto a microtiter plate coated with the murine antibody, mRK35. This capture step was performed at pH 2.5 to ensure myostatin was not complexed to any other serum components to enable quantification of total myostatin levels. Bound myostatin was then detected with a biotinylated murine anti-myostatin antibody biotin-RK22 [27]. An electrochemiluminescence signal was produced by the Meso Scale Discovery SECTOR Imager 6000 (Gaithersburg, MD, USA), using streptavidin-ruthenium and tripropylamine. The measured signal was reported as response units (RU). Sample concentrations were determined by interpolation from a standard curve that was fit using a 4-parameter logistic equation with 1/y2 weighting. Standard curves were prepared using the myostatin-deficient Belgian Blue cow serum. The range of quantification for the monkey assay was 0.0265–100 ng/mL in 10% monkey serum corresponding to 0.265–1000 ng/mL in 100% monkey serum.

### Statistical analysis

Where appropriate, an unpaired two-tailed Student’s *T* test or analysis of variance (ANOVA) with Fisher’s LSD post-test were used. Statistical analyses were performed using Graphpad Prism version 6 (GraphPad Software Inc., San Diego, CA, USA).

## Results

### mRK35 and domagrozumab inhibit myostatin signaling in vitro

In a cell-based myostatin signaling activity assay, both murine (mRK35) and humanized (domagrozumab) anti-myostatin antibodies inhibited human myostatin signaling activity with IC50s < 1 nM. Also, as previously reported, both antibodies were shown to bind to human myostatin with high affinity (mRK35 *K*_*D*_ = 21.8 pM and domagrozumab *K*_*D*_ = 2.6 pM) [21].

The same cell-based signaling assay was also used to examine the effect of mRK35 and domagrozumab on signaling activity induced by other ligands including GDF-11, Activin A, Activin B, and Activin AB, which utilize many of the same receptors as myostatin, and by BMP-9 in a cell-based BMP signaling assay, to understand the specificity and selectivity of our anti-myostatin antibodies. Both antibodies demonstrated a similar selectivity profile in which myostatin and GDF-11 signaling were inhibited at sub-nanomolar concentrations, and inhibition of Activin B was detected but only at higher antibody concentrations. These antibodies did not block signaling induced by Activin A or Activin AB. Also, domagrozumab did not block BMP-9 signaling. This contrasts with the activity of an ActRIIB-Fc soluble myostatin decoy receptor which effectively blocked all ligands tested, at sub-nanomolar levels (Table 1).

**Table 1:** IC_50_ values derived from the cell-based signaling assays

Myostatin inhibitors	IC_50_ with ligand					
	Myostatin	GDF-11	Activin A	Activin B	Activin AB	BMP9
ActRIIB-Fc	< 1 nM	< 1 nM	< 1 nM	< 1 nM	< 1 nM	< 1 nM
Domagrozumab	< 1 nM	< 1 nM	> 1000 nM	81 nM	> 1000 nM	> 1000 nM
mRK35	< 1 nM	< 1 nM	> 1000 nM	37 mM	> 1000 nM	> 1000 nM

Representative IC_50_ values for a minimum of two assays. *ActRIIB-FC* activin receptor IIB-fragment crystallizable, *GDF-11* growth differentiation factor-11

### mRK35 increases body weight and skeletal muscle mass in C57BL/6 and C57Bl/10 mice

**Fig. 1:**
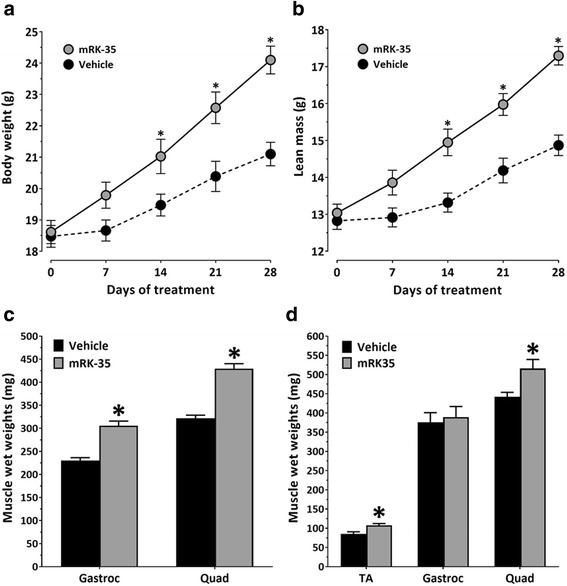
Effects of mRK35 on body weight and skeletal muscle mass in C57BL/6 and C57Bl/10 mice. **a** Weekly body weights following weekly intraperitoneal (IP) administration of 10 mg/kg mRK35 or vehicle (PBS) in 8-week-old male C57Bl/6 mice. **b** Weekly whole body lean mass measured by NMR. **c** Tissue wet weights of the gastrocnemius bundle (Gastroc) and quadriceps (Quad) muscles harvested after 4 weeks of treatment. Both groups (mRK35 and vehicle) contained eight mice. **d** Tissue wet weights of the tibialis antirior (TA), gastrocnemius bundle, and quadriceps of 1-year old male C57Bl/10 mice following weekly IP administration of 10 mg/kg mRK35 or vehicle (PBS) for 4 weeks. Data plotted are means ± SEM. *p < 0.05 versus vehicle as measured by two tailed Student’s *t* test

The effect of mRK35 on skeletal muscle mass was assessed by dosing 8-week-old male C57BL/6 mice IP with 10 mg/kg/week of mRK35 or PBS (vehicle). Within 2 weeks of treatment, the mean body weight in mice receiving mRK35 was significantly increased compared with the vehicle-treated mice. mRK35-treated mice continued to gain body weight above that seen in the vehicle-treated mice for the remainder of the study (Fig. 1a). Weekly body composition measurements by NMR also showed statistically significant increases in lean body mass after 2 weeks of treatment that was maintained throughout the study (Fig. 1b). After 4 weeks of treatment, mice were sacrificed and muscles were collected. The wet weights of the gastrocnemius muscle bundle and quadriceps muscle were significantly increased in the mRK35-treated mice (33 and 34%, respectively) compared with the vehicle-treated mice (Fig. 1c). The effect of mRK35 on skeletal muscle mass in a mature mouse was assessed by dosing 1-year old C57BL/10 mice IP with 10 mg/kg/week of mRK35 or PBS (vehicle). After 4 weeks of treatment, the mice were sacrificed and muscles were collected. The wet weights of the tibialis anterior and quadriceps muscles were significantly increased in the mRK35-treated mice (26 and 17%, respectively) compared with the vehicle-treated mice (Fig. 1d). No significant change in body weight (not shown) or gastrocnemius bundle weight was observed in the older mice.

### mRK35-induced skeletal muscle hypertrophy and improved strength in mdx mice

**Fig. 2:**
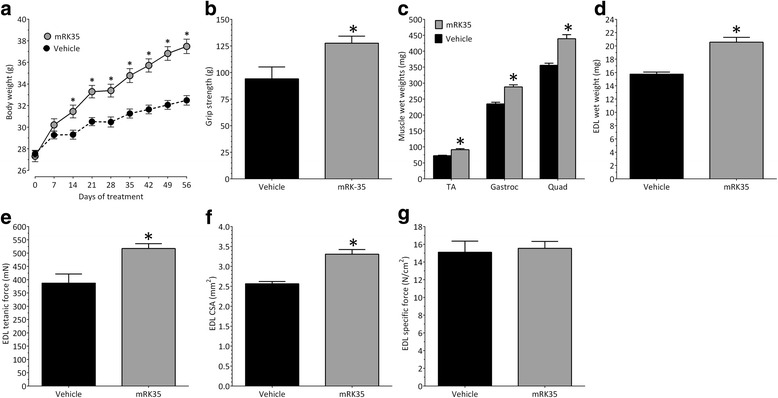
Effect of mRK35 in mdx mice. **a** Weekly body weights following intraperitoneal administration of 10 mg/kg/week mRK35 or vehicle (PBS) in 8-week-old male mdx mice. **b** Forelimb grip strength measured after 8 weeks of treatment. **c** Tissue wet weights of the TA, gastrocnemius, and quadriceps muscles. **d** Tissue wet weight of the EDL. **e** EDL maximum tetanic force from resting length. **f** EDL cross-sectional area. **g** EDL specific force. Treatment groups contained 9 (vehicle) or 10 (mRK35) treated mice. Data plotted are means ± SEM. *p < 0.05 versus vehicle as measured by two-tailed Student’s *t* test

The effect of mRK35 on mdx mice was assessed after dosing 8-week-old male C57Bl/10ScSn-Dmd<*mdx*>/J mice IP with 10 mg/kg/week of mRK35 or vehicle (PBS) for 8 weeks. Body weights were significantly increased after 2 weeks of treatment with mRK35 compared with the vehicle-treated mice, and this effect was maintained throughout the remainder of the study (Fig. 2a). Forelimb grip strength was assessed after 8 weeks of dosing. mRK35 induced a statistically significant increase (21%) over the vehicle-treated group (Fig. 2b). After 8 weeks of treatment, mice were sacrificed and muscles were collected. mRK35 induced a statistically significant increase over the vehicle-treated group in the weight of the tibialis anterior muscle (26%), the gastrocnemius muscle bundle (23%), and quadriceps (23%) (Fig. 2c). The EDL muscle was removed and preserved in oxygenated Ringer’s buffer for ex vivo stimulation. Notably, the wet weight, measured maximum tetanic force, and calculated cross-sectional area of the EDL were each significantly increased by ≥ 29% following 8 weeks of mRK35 treatment (Fig. 2d–f), while no change in specific force was detected (Fig. 2g).

### Increased skeletal muscle fiber size in mRK35-treated mdx mice

**Fig. 3:**
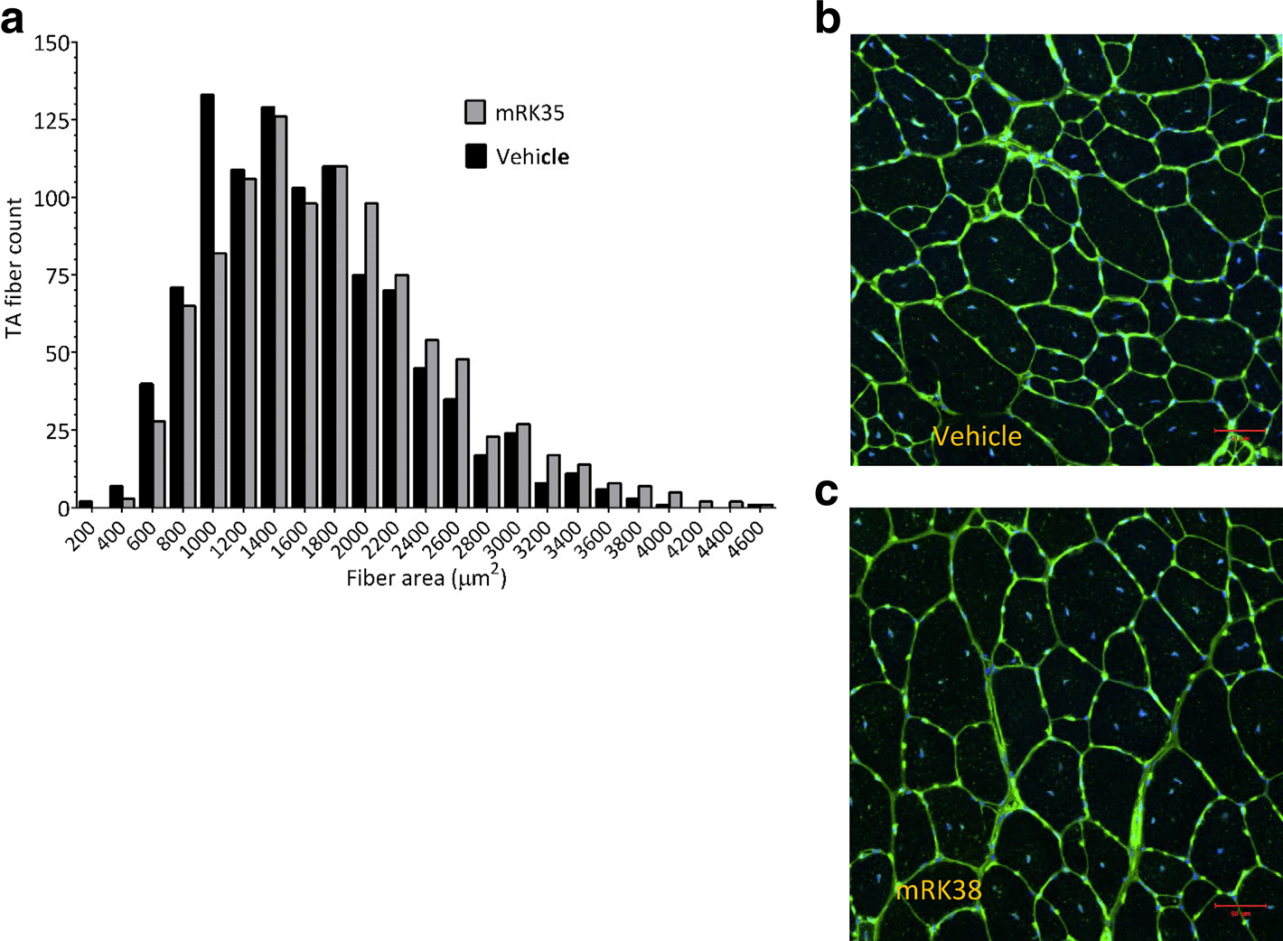
Effect of mRK35 on skeletal muscle fiber size in mdx mice. **a** Fiber area count at the mid-belly of the TA muscle harvested from mdx mice following 4 weeks of treatment with 10 mg/kg mRK35 or vehicle (*n* = 4 per treatment). **b** Representative cross-sectional images of the TA from vehicle-treated mice. **c** Representative cross-sectional images of the TA from mRK35-treated animals. Muscle fibers are stained with wheat germ agglutinin in green and counterstained with DAPI, which stains the nuclei, in blue

An increase in TA fiber area was seen in mdx mice after 4 weeks of treatment with 10 mg/kg/week mRK35, compared with vehicle-treated mdx mice (Fig. 3a). This was quantified as a significant increase of 12.4 ± 2.2% in the mean fiber diameter in the mRK35-treated versus vehicle-treated mdx mice, while fiber number per muscle remained unchanged across treatments. Representative images of TA fiber sections from the vehicle (Fig. 3b) and 10 mg/kg mRK35-treated group (Fig. 3c) are shown with wheat germ agglutinin (muscle membrane) and DAPI (nuclei) stains. No change in the number of centrally nucleated fibers was noted.

### Domagrozumab, humanized anti-myostatin antibody, induced skeletal muscle hypertrophy in cynomolgus monkeys

**Fig. 4:**
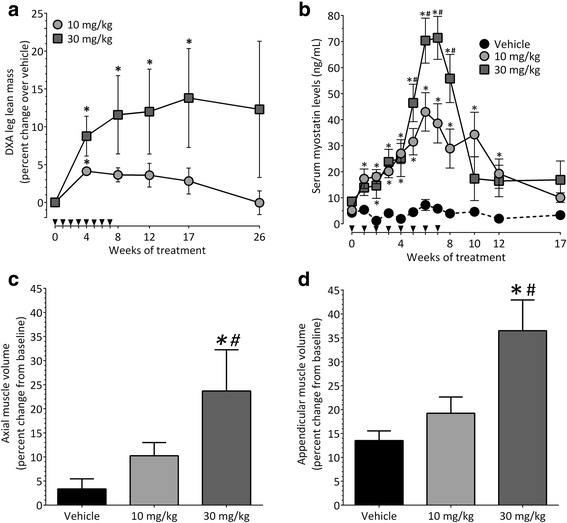
Effect of domagrozumab on skeletal muscle in cynomolgus monkeys. **a** Percent change in leg lean muscle mass over vehicle (PBS), measured by DXA, following 8 weeks of treatment with 10 and 30 mg/kg domagrozumab. **b** Myostatin levels in serum collected from all monkeys. **c** Percent change from baseline (start of study) in axial muscle volume after 8 weeks of treatment. **d** Percent change from baseline in appendicular muscle volume after 8 weeks of treatment. *N* = 5 animals for vehicle and 10 mg/kg groups and 3 animals for 30 mg/kg group. Data plotted are means ± SEM. * denotes *p* < 0.05 via ANOVA with Fisher’s LSD post-test versus vehicle; # denotes *p* < 0.05 versus 10 mg/kg domagrozumab; denotes time of dosing

The effect of domagrozumab on cynomolgus monkeys was assessed after weekly IV dosing of 10 or 30 mg/kg domagrozumab or vehicle (PBS) for 8 weeks. By week 4 of treatment with domagrozumab, DXA assessment of leg muscle mass indicated a statistically significant increase in both the low- (10 mg/kg, 4.8 ± 0.4%) and high- (30 mg/kg, 9.4 ± 6.0%) dose groups compared with the vehicle group. In the low-dose group, statistical significance associated with the increase in muscle mass was not maintained following the cessation of treatment (Fig. 4a). However, in the high-dose group, the statistically significant increase in muscle mass was maintained until week 17, which was 10 weeks beyond administration of the last dose.

To demonstrate target engagement, serum samples were analyzed for myostatin modulation. In the vehicle-treated monkeys, myostatin levels remained unchanged between week 0 and 17 of the study. A dose-dependent increase in myostatin level was seen in the domagrozumab-treated groups (Fig. 4b). Statistical significance for the increase in myostatin serum levels over vehicle-treated monkeys was seen after 2 weeks of treatment with the 10 mg/kg dose and after 3 weeks with 30 mg/kg dose. Also, elevated serum myostatin levels were observed in the 10 mg/kg dose group until week 12, 5 weeks beyond administration of the last dose (Fig. 4b).

To examine the effect of domagrozumab on skeletal muscle volume, CT scans of the axial and appendicular muscles were collected at baseline and at week 8. In the vehicle-treated group, mean axial muscle volume increased 3.3 ± 4.7% by week 8, as compared with increases of 10.2 ± 6.2 and 23.7 ± 16.9% in the 10 and 30 mg/kg dose groups, respectively. The increase in mean axial muscle volume of the high-dose group achieved statistical significance over the change in volume of both the vehicle and low-dose groups (Fig. 4c). A similar trend was observed in the appendicular muscles, with the vehicle group showing a 13.5 ± 4.5% increase by week 8, and increases of 19.2 ± 7.6 and 36.5 ± 20.3% for the low- and high-dose groups, respectively. Again, the increase in mean appendicular muscle volume of the high-dose group achieved statistical significance over the change in volume of both the vehicle and low-dose groups (Fig. 4d). No muscle force measurements were performed on the monkeys.

## Discussion

Due to muscle atrophy and the associated loss of ambulation, minimizing muscle wasting and/or maintaining muscle mass and function remains a viable treatment strategy for DMD patients. Even with the conditional approval of two potentially disease-modifying therapies for DMD patients (ataluren and eteplirsen), and the development of gene therapy approaches to address the primary defect in this disease (dystrophin deficiency), there remains a need to retain intact muscle fibers for the translation of functional dystrophin protein. Pharmacological inhibition of myostatin has the potential to induce increased muscle mass and strength, which may prove to benefit patients as a stand-alone therapy or in combination with disease-correcting approaches.

Supporting the hypothesis that myostatin inhibition will convey a functional benefit to DMD patients, at least two other myostatin inhibitor drug candidates are in clinical testing for DMD: BMS-986089, a myostatin adnectin (ClinicalTrials.gov identifier# NCT02515669) and rAAV1.CMV.huFollistatin344, follistatin gene therapy, (ClinicalTrials.gov identifier# NCT02354781). Other myostatin inhibitors have been tested in patients with different muscle wasting conditions including sarcopenia, cachexia, and adult muscular dystrophies [28], but none have demonstrated the required efficacy in functional endpoints to allow regulatory approval. The first myostatin inhibitor to be administered to DMD patients was ACE-031, a fusion protein of ActRIIB and IgG1-Fc. Although the clinical trial of this compound was stopped due to safety findings, trends for increased lean body mass and maintenance of the 6-min-walk test were seen [29]. These findings suggested that modulation of myostatin signaling in DMD patients may provide a functional benefit. In previous clinical trials with a selective anti-myostatin antibody, MYO-029, no improvement in endpoints related to muscle strength and function was seen in adult muscular dystrophy patients [30]. Although DMD patients were not included in this trial, the findings raised questions about the future application of a selective myostatin antibody to this rare disease population. However, retrospective pharmacokinetic/pharmacodynamics assessment of MYO-029 dosing in this study suggested that insufficient target coverage in the patient cohorts may have contributed to the lack of robust increases in muscle mass and strength [31]. Further, in preclinical studies, mRK35 significantly improved muscle strength compared to MYO-029 [21]. Importantly, improved target coverage has been demonstrated in healthy subjects dosed with domagrozumab up to 40 mg/kg via IV infusions, thereby providing increased confidence in the likelihood of achieving efficacy in DMD patients with this next-generation antibody [32].

In this report, mRK35 and its humanized equivalent, domagrozumab, demonstrated potent inhibition of myostatin signaling in a cell-based reporter assay. This assay also demonstrated the selectivity of these antibodies as shown by minimal or no inhibition of several related ligands, including Activins A, B, and AB. Furthermore, domagrozumab did not inhibit BMP-9-induced signaling in a similar cell-based assay designed to examine modulation of BMP signaling (Table 1). By comparison, ActRIIB-Fc demonstrated potent inhibition of all ligands tested, including BMP-9. Importantly, the off-target inhibition of BMP-9 by ACE-031 was associated with adverse events in DMD patients, which included observation of epistaxis and telangiectasias [33]. Therefore, it is anticipated that the greater selectivity and lack of BMP-9 inhibition seen with domagrozumab will result in an improved clinical safety profile over ACE-031.

Our in vivo assessment of mRK35 in wild-type and mdx mice demonstrated the efficacy of mRK35 as shown by increased lean body mass and individual skeletal muscle weights (Figs. 1 and 2). This was associated with increased grip strength and maximum tetanic force in both wild-type and mdx mice (Fig. 2) [21]. Increased muscle fiber cross-sectional area, with no change in fiber number, was also seen in the mdx mouse muscle samples collected from the mRK35-treated group (Fig. 3). Although not quantified here, there was no apparent effect of mRK35 treatment on the proportion of fibers with centralized nuclei, suggesting that mRK35 did not promote muscle regeneration (Fig. 3b, c). This observation is consistent with previous work with mdx mice treated with a myostatin antibody that also showed no change in the number of regenerating fibers in the mdx mouse diaphragm [34]. Our examination of the histopathology of the TA and diaphragm muscle samples found a relatively low level of fibrosis in the vehicle-treated mdx mouse muscles (data not shown). This may be due to the age of the mdx mice in these studies. Therefore, an assessment of the anti-fibrotic effects of mRK35 was not undertaken. In previous studies, however, inhibition of myostatin in mdx mice has resulted in improvements in fibrosis in the diaphragm. Additional longer treatment studies are in progress to examine effects of mRK35 on fibrosis that is evident in the diaphragm and skeletal muscle of older mdx mice and the more severe DBA2-mdx mouse model. Also, in 1-year old wild-type mice which have reduced circulating myostatin levels [35], treatment with mRK35 still significantly increased skeletal muscle mass. In non-human primates, target engagement of the humanized myostatin antibody was demonstrated with weekly measures of serum myostatin that showed a dose-dependent increase in circulating myostatin levels over the 8-week domagrozumab dosing period (Fig. 4b). In parallel to these measures of serum myostatin, the pharmacological effect of inhibiting myostatin signaling was evaluated by longitudinal DXA measures that found a dose-dependent increase in lean mass in the legs of monkeys receiving domagrozumab (Fig. 4a). In addition, CT assessment of skeletal muscle found a corresponding increase in muscle volume of both axial and appendicular sites.

There are concerns within the DMD community about the potential for detrimental effects that myostatin blockade may have on patients. The principle concern is the possibility of myostatin inhibition exacerbating existing muscle contractures caused by the imbalance of muscle hypertrophy and atrophy in DMD. Indeed, Kornegay et al. [36] reported GRippet dogs, which are myostatin heterozygous whippets crossed with the GRMD (golden retriever muscular dystrophy) dog, have increased contractures likely caused by unequal muscle hypertrophy and atrophy as shown by MRI. It is possible the GRippet dogs’ reduced myostatin levels during development may have influenced the contracture incidence, but this is not known. Bish et al. [37] did not report any contracture changes in GRMD dogs with AAV expression of a dominant negative myostatin peptide despite unequal skeletal muscle hypertrophy, but it is unknown if joint angles were looked at in this study. As such, this question remains unanswered and should be addressed.

Another concern with myostatin inhibition in DMD is around the potential for increased damage due to increased force production from muscle hypertrophy. Here, Bish et al. [37] demonstrated AAV expression of a dominant negative myostatin peptide led to increased skeletal muscle mass and reductions in circulating creatine kinase and fibrosis, indicating that in at least the dog model, this is not the case.

Finally, there are concerns that the increased muscle hypertrophy driven by prolonged myostatin inhibition will be deleterious to the muscle satellite cell population already tasked with repairing the continual damage caused by the disease. As reported by Kornegay et al. [36], GRippet dogs have no discernable change in satellite cell pool number or indications of satellite cell exhaustion at 3 years of age despite decreased circulating myostatin levels, muscle hypertrophy, and a severe muscular dystrophy phenotype. These finding are especially significant given that dogs have similar telomere length and activity as compared to humans [38].

While it is certainly important to keep all these concerns in mind, we believe the demonstrated benefits of myostatin blockade both in preclinical models of DMD and even in DMD patients receiving a non-selective myostatin inhibitor [29] outweigh the potential risks of further testing this more selective myostatin antibody. In addition to the benefits of myostatin blockade in NHPs and in wild-type and mdx mice detailed here and elsewhere [15–20], there are multiple studies in dogs, both healthy [39] and dystrophic [36], demonstrating the positive effects of myostatin blockade.

## Conclusions

In this report, we provide pharmacology data that demonstrates the muscle anabolic activity of the anti-myostatin antibody mRK35 and its clinical analog domagrozumab (PF-06252616) in rodents, including the mdx mouse model of DMD, and non-human primates. These findings have provided justification to further develop this antibody drug candidate for the treatment of DMD patients. A recently completed Phase 1 study with this compound has demonstrated an appropriate safety and tolerability profile in healthy volunteers [32], and a Phase 2 clinical trial in DMD patients is currently underway (ClinicalTrials.gov identifier #NCT02310763).

## Abbreviations

AAALAC: Association for Assessment and Accreditation of Laboratory Animal
ActRIIB: Activin receptor type IIB
ANOVA: Analysis of variance
BMP: Bone morphogenic protein
BRE: BMP-response element
CHO: Chinese hamster ovary
CT: Computerized tomography
DAPI: 4′,6-Diamidino-2-phenylindole
DBPS: Dulbecco’s phosphate buffered saline
DMD: Duchenne muscular dystrophy
DMEM: Dulbecco’s Modified Eagle’s Medium
DXA: Dual energy X-ray absorptiometry
EDL: Extensor digitorum longus
ELISA: Enzyme-linked immunoassay
EU: European Union
GDF-8: Growth differentiation factor-8
GRMD: Golden retriever muscular dystrophy
IACUC: Institutional Animal Care and Use Committee
IP: Intraperitoneal
mRK35: Murine RK35
NMR: Nuclear magnetic resonance
RGA: Reporter gene assay
RU: Response unit
TA: Tibialis anterior
TGF-β: Transforming growth factor-β
USA: United States of America
